# The Accuracy and Precision of Gait Spatio-Temporal Parameters Extracted from an Instrumented Sock during Treadmill and Overground Walking in Healthy Subjects and Patients with a Foot Impairment Secondary to Psoriatic Arthritis

**DOI:** 10.3390/s21186179

**Published:** 2021-09-15

**Authors:** Roua Walha, Karina Lebel, Nathaly Gaudreault, Pierre Dagenais, Andrea Cereatti, Ugo Della Croce, Patrick Boissy

**Affiliations:** 1 Faculty of Medicine and Health Sciences, Université de Sherbrooke, Sherbrooke, QC J1H 5N4, Canada; Walha.Roua@usherbrooke.ca (R.W.); Nathaly.Gaudreault@usherbrooke.ca (N.G.); Pierre.Dagenais@usherbrooke.ca (P.D.); 2 Research Center on Aging, CIUSSS Estrie CHUS, Sherbrooke, QC J1H 4C4, Canada; Karina.Lebel@usherbrooke.ca; 3 Faculty of Engineering, Université de Sherbrooke, Sherbrooke, QC J1K 2R1, Canada; 4 Department of Electronics and Telecommunications, Politecnico di Torino, 10129 Torino, Italy; andrea.cereatti@polito.it; 5 Department of Biomedical Sciences, University of Sassari, 07100 Sassari, Italy; dellacro@uniss.it; 6 Biomedical Engineering Department, Catholic University of America, Washington, DC 20064, USA

**Keywords:** wearable systems, IMUs, gait parameters, free-living measures

## Abstract

The objectives of this study were to assess the accuracy and precision of a system combining an IMU-instrumented sock and a validated algorithm for the estimation of the spatio-temporal parameters of gait. A total of 25 healthy participants (HP) and 21 patients with foot impairments secondary to psoriatic arthritis (PsA) performed treadmill walking at three different speeds and overground walking at a comfortable speed. HP performed the assessment over two sessions. The proposed system’s estimations of cadence (CAD), gait cycle duration (GCD), gait speed (GS), and stride length (SL) obtained for treadmill walking were validated versus those estimated with a motion capture system. The system was also compared with a well-established multi-IMU-based system for treadmill and overground walking. The results showed a good agreement between the motion capture system and the IMU-instrumented sock in estimating the spatio-temporal parameters during the treadmill walking at normal and fast speeds for both HP and PsA participants. The accuracy of GS and SL obtained from the IMU-instrumented sock was better compared to the established multi-IMU-based system in both groups. The precision (inter-session reliability) of the gait parameter estimations obtained from the IMU-instrumented sock was good to excellent for overground walking and treadmill walking at fast speeds, but moderate-to-good for slow and normal treadmill walking. The proposed IMU-instrumented sock offers a novel form factor addressing the wearability issues of IMUs and could potentially be used to measure spatio-temporal parameters under clinical conditions and free-living conditions.

## 1. Introduction

Efficient and stable gait is an indicator of autonomy and good health. In some inflammatory arthropathies such as psoriatic arthritis (PsA), the frequency and severity of foot and ankle problems such as synovitis, tendonitis, enthesitis, etc., could result in high levels of pain, deformities, and a reduced range of motion. Consequently, antalgic gait strategies may appear and disrupt the cyclical and symmetric process of normal gait. In such cases, an objective gait analysis enables clinicians and researchers to monitor disease progression and patient responses to interventions such as rehabilitation and orthotics. Spatio-temporal gait parameters, among other variables such as joint kinematics, kinetics, and electromyography are usually used to quantify changes/alterations in gait patterns and are key metrics in gait analysis. The main spatio-temporal parameters including stance, swing, and stride duration, also named gait cycle duration (GCD), cadence (CAD), stride length (SL), and gait speed (GS) are important clinical outcomes as they allow the functional status to be described and adverse health outcomes to be predicted [1]. For instance, a reduced GS is associated with functional independence [2] and is used as a screening tool for foot pain [3]. SL is also considered as an indicator of foot pain [4] and the severity of rheumatological and musculoskeletal disorders (associated with a reduction in the ankle, knee, or hip range of motion) such as osteoarthritis [5]. On the other hand, temporal gait parameters such as GCD, stance duration, and swing duration are used as indicators of gait instability, risk of falling, and frailty syndrome [6]. Simple methods such as the stopwatch can be used to measure basic parameters such as GS in clinical settings. Laboratory-based methods, such as force platforms, gait mats, and motion capture (Mocap) systems are used as gold standards to measure spatio-temporal parameters in research settings [7]. Laboratory-based methods are highly accurate but expensive, may require evaluator expertise, limit the assessment to short time intervals and to the laboratory space, and thus may not be representative of the participants’ real-life walking patterns [8]. For these reasons, wearable systems based on Inertial Measurement Units (IMUs) have been developed and extensively used in the last two decades for gait analysis both in research and in clinics [9].

Wearable IMUs are miniature movement sensors usually composed of a three-axis accelerometer and a three-axis gyroscope, often integrated with a three-axis magnetometer. Several systems using one or more IMUs that can be placed on different body segments are available for clinical use and research purposes. Among them, the Mobility Lab (ML) system (APDM^®^, Portland, OR, USA), uses a set of six IMUs worn on the chest, lower back, wrists, and feet and comes with software that allows for an automated estimation of several gait parameters. ML is widely used in clinics and research [10] and has been validated in healthy [11,12] and pathological populations [13,14,15]. However, such a system remains costly and due to the number and placement of IMUs, it is not always practical and usable in clinics and certainly not for free-living gait recordings.

Independently of their ease of use and accessibility, questions about the accuracy and precision of the spatio-temporal parameter data derived from IMUs still remain as they depend on a combination of factors including sensor noise and drift, environmental perturbations, the type and specific tuning of the orientation’s estimation filter [16], the positioning and alignment of sensors, as well as the level of complexity of the processed parameters [9]. Indeed, the accuracy of temporal gait parameters mainly depends on the ability of the system to accurately detect gait events (GEs). The estimation of the accuracy of spatial parameters, however, depends upon a series of factors including the accuracy of GE detection, the accuracy of orientation estimations, and the ability of the algorithm to correct integration errors. As mentioned above, the first step to estimate spatio-temporal parameters from IMU recordings requires the accurate detection of GEs specifically, namely initial contact (IC) or heel strike, and final contact (FC) or toe-off. Several methods based on angular velocities or accelerations from one or more IMUs have been proposed in the literature to detect GE and to compute the temporal parameters [9]. However, the accuracy of the GE detection can vary drastically depending on the sensors used, their placement, the recording used (e.g., acceleration and angular velocity), the algorithm used, and the study population. A recent systematic review [9], showed that foot and shank/ankle-based algorithms have a better accuracy and repeatability than wrist or lower trunk-based ones and that angular velocity-based algorithms are more accurate than those based on acceleration signals for GE detection and the estimation of temporal parameters.

Validation studies of foot-mounted IMUs for the measurement of spatio-temporal parameters have shown varying results [17,18,19]. For example, a study by Donath et al. showed a good to excellent intersession reliability and validity for the commercial Rehagait system in the estimation of spatio-temporal parameters in a sample of healthy participants (N = 22) for treadmill walking at different speeds including slow (0.95 m/s), normal (1.12 m/s), and fast (1.28 m/s) and slopes (0% and 15%). The results showed a lower reliability and validity for the estimates at slow speeds and a 15% slope; however, the average relative errors for SL, GCD, and CAD were as small as 2.7%, 0.8%, and 1.5%, respectively, for all speeds and slopes compared to data from an instrumented treadmill [17]. Rampp et al. also validated a shoe-mounted IMU (Shimmer 2R) in 116 older adults during overground walking. Their findings showed a good agreement between the IMU system and an instrumented mat (GAITRite). A mean absolute error (MAE) of less than 33 ms and 6.3 cm was found for the temporal parameters and SL, respectively [18]. However, in another study, the findings showed a poor concurrent validity for the Rehawatch system (foot-mounted IMUs–WS) compared to the instrumented mat in 21 frail older adults, especially at fast walking [19].

The validity and reliability of several ankle–IMU-based methods have also been studied and most of the studies reported encouraging results [20,21,22]. For instance, Trojaniello et al. proposed and validated a method named the Trusted Events and Acceleration Direct and Reverse Integration along the direction of Progression (TEADRIP) using two ankle-mounted IMUs (Opal, APDM) for the determination of the spatio-temporal parameters in hemiparetic, choreic, and Parkinson’s disease patients and healthy older adults [22]. Compared to the instrumented mat (GAITRite) data, the mean errors in IC detection in all groups ranged from 0 to 11 ms (MAE: 10 to 17 ms) for normal speed walking, and from 3 to 22 ms (MAE: 12 to 22 ms) for fast walking. The relative errors in the SL estimation ranged from 1 to 3% in all groups and for both speeds [22]. The TEADRIP was also validated in a multicenter study in a large number of participants with and without PD and mild cognitive impairment (N = 236) [21]. The results showed an average delay of 10 ms in IC identification performed by the TEADRIP in all clinical centers and for all walking speeds. The relative errors for both the temporal and spatial parameters were below and never over 3%. The accuracy of estimating the temporal gait parameters for this same method was investigated in the study by Storm et al. for free-living walking using shank-mounted IMUs in a sample of ten healthy participants [20]. Their results showed reasonable MAE values of about 9 and 13 ms in stride time and step time estimations, respectively, but a higher MAE for stance time (37 ms).

In summary, one can presume that foot and shank/ankle-based methods have comparable accuracy and reliability and amongst the different methods, the TEADRIP method applied to ankle IMUs seems to be robust for the calculation of spatio-temporal parameters and so far, it is the only method that has been validated in a large number of participants and different populations such as healthy, Parkinson’s disease, hemiparetic, and choreic patients. However, this method, or any other IMU-based method, has never been assessed in patients with foot impairments secondary to PsA for whom the presence of foot pain may result in a specific antalgic gait pattern different from that observed in neurological conditions. Moreover, in terms of usability, the studies suggest that shank-mounted IMUs could be less cumbersome and be preferred over foot-mounted ones, which is an important consideration for long and free-living gait recordings [23,24]. Advances in the miniaturization and packaging of IMUs and their integration in garments (or the so-called smart textile systems) could offer solutions to enhance the wearability and comfort of IMUs while allowing them to stay close to the skin with a relatively fixed position [25]. Among them, a low-cost system consisting of an instrumented sock including an ankle-mounted IMU (Sensoria ^®^) is now available on the market [26].

The purpose of this study was to assess and compare the accuracy (concurrent validity) and precision (intersession reliability) of the spatio-temporal parameters estimated with the proposed system applying the TEADRIP algorithm to the above mentioned IMU-instrumented sock recordings in HP and PsA patients with foot problems.

## 2. Materials and Methods

### 2.1. Subjects

Twenty-five healthy participants (HP) were recruited (10 males, 15 females). The mean age and body mass index of the HP were 31.08 ± 10.15 years and 24.6 ± 4.3, respectively. The inclusion criteria included those aged 20 years or older. The exclusion criteria included those suffering from lower limb pain or any musculoskeletal, rheumatological, or neurological disease that could affect normal gait patterns. Twenty-one PsA patients (5 males, 16 females) with a mean age of 53.9 (8.9) years, a BMI of 29.3 (4.5), and a mean disease duration of 11.5 years (10.2) were consecutively recruited from the rheumatology out-patient clinics at the Hotel Dieu University Hospital CHU of Sherbrooke (CHUS). The inclusion criteria were as follows: aged between 20 and 70 years, a confirmed diagnosis of PsA by a trained rheumatologist, moderate to severe and recurrent foot pain, and stable medication over three months preceding the recruitment. The exclusion criteria included patients with diabetes, neurological disease, or any musculoskeletal disease that could impact normal gait patterns. The study was approved by the CIUSSS de l’Estrie-CHUS ethics committee, and all the participants gave their informed consent.

### 2.2. Study Design

This study is part of a quasi-experimental trial that aims to explore the effectiveness of custom-made foot orthotics on pain, function, and clinical and ecological gait parameters in PsA patients.

**Figure 1:**
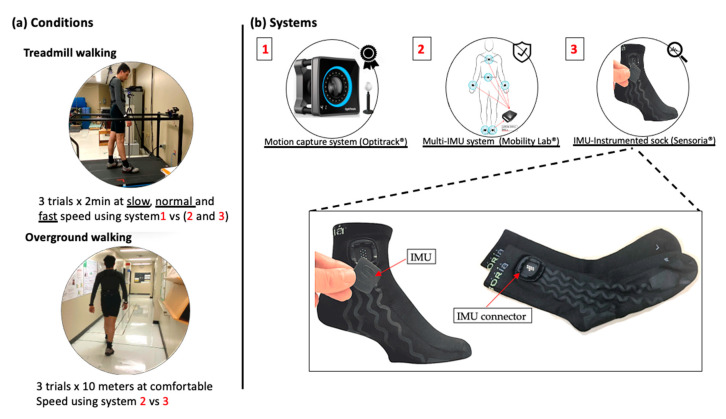
Conditions and measurement systems used for data acquisition. (**a**) Participants performed three 2 min treadmill walking trials at slow, normal, and fast walking speeds and three 10 m overground walking trials at a self-selected comfortable speed. (**b**) A motion capture system was used as the gold standard to validate the estimations of the spatio-temporal parameters for treadmill walking based on the IMU-instrumented sock and the Mobility Lab system.

Two walking conditions, treadmill and overground walking, were considered (Figure 1a). An optical motion capture system was used to obtain spatio-temporal parameter values to be used as the gold standard for treadmill walking, while for overground walking, reference values for the spatio-temporal parameters were obtained from the Mobility Lab system (APDM Wearable Technologies, Portland, OR, USA). HP attended two measurement sessions that were one week apart, while PsA patients attended one measurement session. The measurement sessions were conducted in the biomechanical laboratory at the research center on aging. The treadmill tests consisted of three 2 min walking trials performed at slow, normal, and fast speeds and recorded using the motion capture system, the IMU-instrumented sock, and the ML system. For HP, slow, normal, and fast speeds were set at 0.45, 1.12, and 1.6 m/s, respectively. For PsA patients, the slow speed was fixed at 0.45 m/s while the normal and fast speeds were determined through a trial-and-error approach where the normal and fast speeds were fixed for each participant as the most comfortable and the fastest possible walking speed, respectively. The overground walking consisted of three trials of the timed 10 m walking tests (10 MWT), a well-known clinical test [27]. The gait data were recorded using the IMU-instrumented sock and the ML system. A stopwatch was also used to record the walking time to compute a clinical standard value of GS.

### 2.3. Measurement Systems

Figure 1b illustrates the measurement systems used for data acquisition. The motion capture system used in this study is composed of eight Optitrack Prime 13W cameras (NaturalPoint, Corvallis, OR, USA). The cameras were calibrated before every data collection using the CWM-125 calibration wand. A computer-controlled treadmill was placed in the center of the volume captured by the cameras. Participants were instrumented using 16 passive markers and a conventional lower body model was used for analysis. Spatio-temporal parameters from the kinematic data were calculated by first identifying GE from the horizontal position of the heel marker as described in [28]. CAD is defined as the number of steps per minute, GCD as the time elapsed between two consecutive heel strikes (HS) of the same foot, SL as the distance between two consecutive HS of the same foot, and GS as SL divided by GCD. Based on these definitions, the average temporal and spatial parameters were then calculated.

The IMU-based system used for this study was the Mobility Lab (ML) system (APDM Wearable Technologies, Portland, OR, USA), a research-grade system widely used by researchers and clinicians for gait and balance analysis. ML is composed of a set of one to six IMUs (Opal, a three-axis accelerometer, a three-axis gyroscope, and a three-axis magnetometer), an Access Point for wireless data transmission and synchronization, and software that provides the estimation of several spatio-temporal parameters including CAD, GCD, GS, and SL. Details on the calculation of the spatio-temporal parameters with the Mobility Lab system are provided in [29]. In this study, we used a setup of six IMUs placed on both wrists, the feet, the lower back, and the chest, as recommended. The sample rate frequency was set at 128 Hz.

The IMU-instrumented sock was manufactured by Sensoria (Sensoria Inc, Redmond, WA, USA) and included an IMU positioned about 5 cm above the lateral malleolus (Figure 1b). To ensure acceptability and limit the potential discomfort due to wearing the sock, participants wore only one IMU-instrumented sock on the right foot during the experiments. Moreover, the IMU’s sampling frequency was limited at 50 Hz to extend the battery life. The IMU data was transferred via Bluetooth to a smartwatch (Apple Watch, series 3), stored, and transferred to a PC via Wi-Fi. The recordings were then processed with a Matlab implementation of the TEADRIP algorithm for the detection of gait events and the estimation of the spatio-temporal parameters [22]. The IMUs were first calibrated to minimize any noise that may affect the detection algorithm. The TEADRIP algorithm, originally developed for bilateral IMUs, was slightly modified to operate on a single IMU to first identify time intervals of interest, corresponding to a first approximation of the swing and stance phases of each step, based on an analysis of the variation in angular velocity. A further analysis of the angular velocity within these specific time intervals identifies the gait events (HS and TO). Specifically, HS corresponds to the local minimum of the medio-lateral angular velocity preceding maximum antero-posterior acceleration. TO is located at the absolute minimum in antero-posterior acceleration occurring prior to the last maximum in antero-posterior acceleration within the specified research window. Using these GEs, the temporal parameters can be obtained. Spatial parameters require the estimation of a change in position. This is performed using a direct and reverse double-integration principle on linear acceleration over the direction of progression. With SL assessed, GS can be estimated in a continuous matter.

### 2.4. Acquisition Protocol

Participants performed three walking trials on the treadmill while simultaneously wearing the IMU-instrumented sock, the ML system IMUs, and the motion capture system’s retroreflective markers. Trials were two minutes long and were carried out at slow, normal, and fast speeds as described previously. The speeds were administered in a balanced order across participants using a Latin square design table. A 30 s warm-up period was given to the participants at the beginning of each trial. All participants performed three trials of the 10MWT wearing the IMU-instrumented sock on the right foot with the sensor positioned above the lateral malleolus and the ML system IMUs. Participants were asked to walk at a comfortable speed along a 14 m straight walkway. To exclude the acceleration and deceleration phases at the beginning and the end of the trial, only the data recorded while walking in the central 10 m of the walkway were considered.

The 10MWT and the treadmill walking tests were repeated at two measurement sessions a week apart for the HP, and only once for the APSO. Data acquisition was performed by the same evaluators within and between the measurement sessions.

### 2.5. Statistical Analysis

The results are reported as means and standard deviations when applicable. The independent *t*-test was used to assess the differences in the spatio-temporal parameters measured for overground walking between healthy and PsA participants. The accuracy (concurrent validity) of the gait spatio-temporal parameters estimated with the TEADRIP method using data from the IMU-instrumented sock recordings and those estimated with the ML system were evaluated against the motion capture system estimates using the Bland–Altman plots. The mean differences between the systems were calculated, were referred to as the bias, and the 95% limits of agreement were determined. The MAE and relative errors (%) derived from the TEADRIP estimations applied to the IMU-instrumented sock recordings and from the ML system estimations were calculated and compared using a two-factor ANOVA for repeated measures and Bonferroni post hoc tests were computed when main effects were detected. A two-way ANOVA was conducted to examine if the effect of the systems on MAE (instrumented sock vs. Mobility Lab) differed between the groups (HP vs. PsA). The precision (intersession reliability) of the TEADRIP estimations from the IMU-instrumented sock recordings and the estimations from the ML system was calculated with Intra Class Correlation Coefficients (ICCs) using a two-way mixed-effects model for absolute agreement (ICC (3, 1)). ICCs values of less than 0.5, between 0.5 and 0.75, between 0.75 and 0.9, and greater than 0.9 were considered as indicators of poor, moderate, good, and excellent reliability, respectively. Statistical analyses were performed under SPSS Version 26 (IBM statistics Corporation, Armonk, NY, USA).

## 3. Results

Cadence, GCD, GS, and SL estimated with the TEADRIP algorithm applied to the IMU-instrumented sock and those estimated with the Mobility Lab system for overground walking were statistically different between the groups (Table 1). PsA patients had a lower CAD, GS, and SL and a higher GCD compared to the healthy participants (*p* < 0.05). For treadmill walking, the mean normal and fast speeds in PsA patients were 1.01 m/s (0.17) and 1.31 m/s (0.21) vs. 1.12 m/s and 1.6 m/s in the HP.

**Figure 2:**
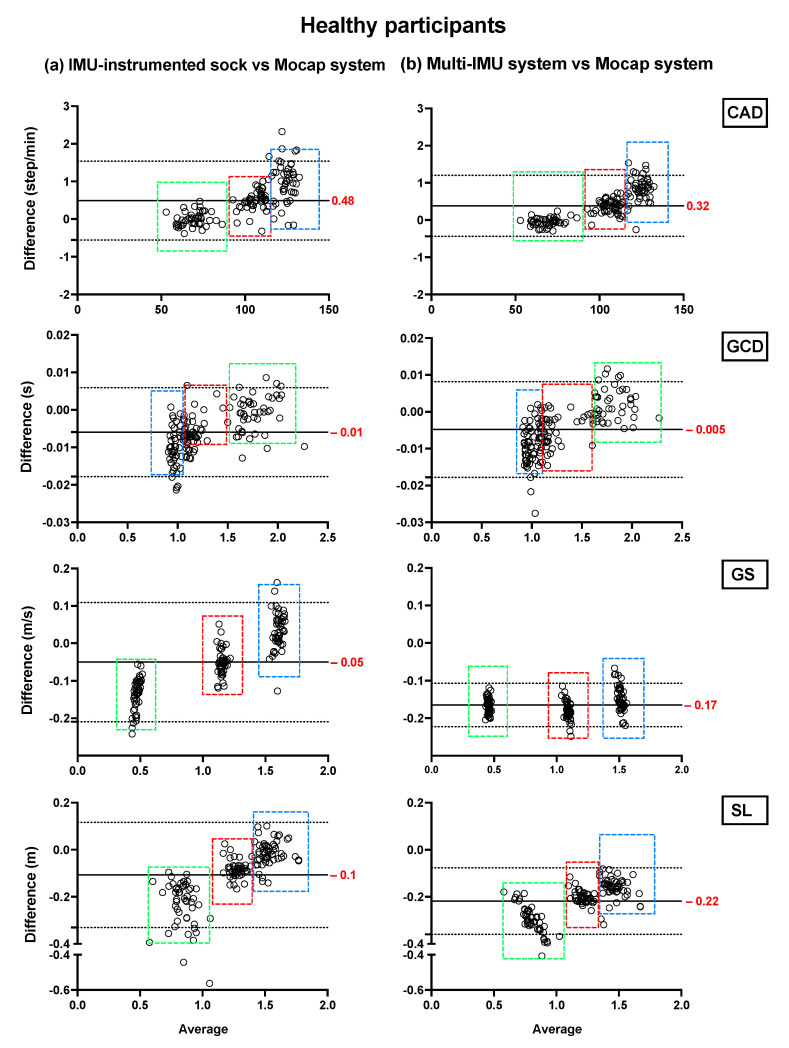
Concurrent validity. Bland–Altman plots of the differences between (**a**) the TEADRIP algorithm applied to the IMU-instrumented sock recordings and the Mocap system, and between (**b**) the Mobility Lab system and the Mocap system during two-minute treadmill walking at different speeds in HP (n = 25, 2 sessions). Cadence (CAD), gait cycle duration (GCD), gait speed (GS), stride length (SL), and Motion capture system (Mocap). The solid lines indicate the mean test-retest differences (bias) and the dashed lines indicate the upper and lower 95% limits of agreement (1.96 SD of the bias). Dashed green, red, and blue squares represent the observations for slow, normal, and fast speeds, respectively.

**Figure 3:**
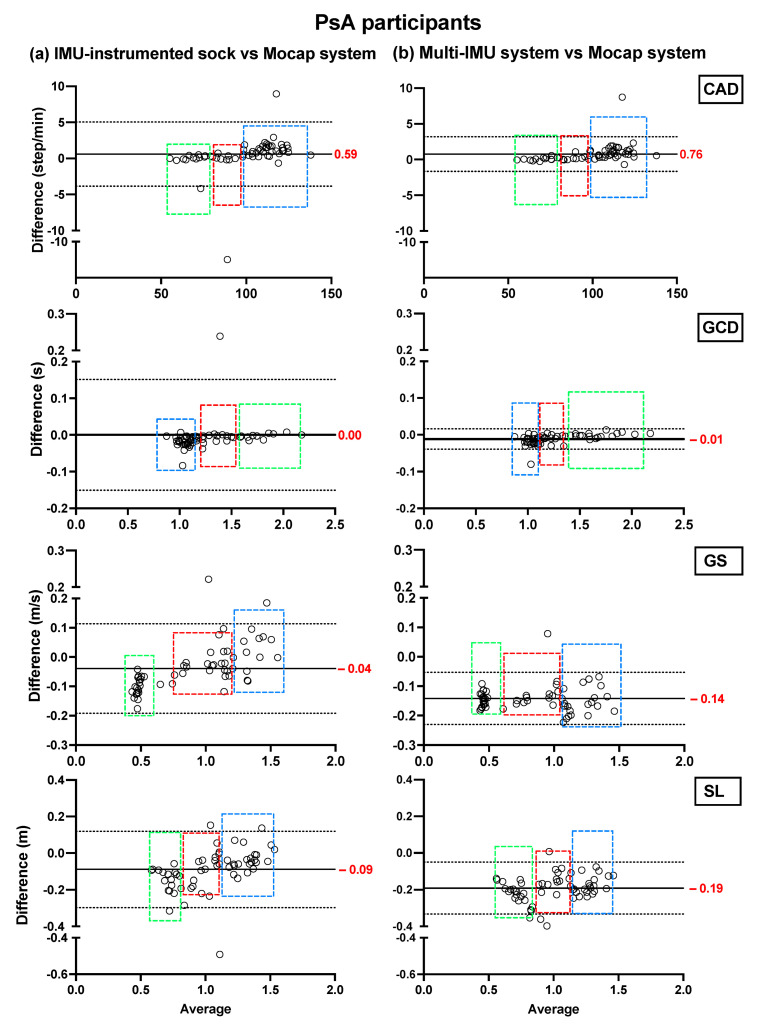
Concurrent validity. Bland–Altman plots of the differences between (**a**) the TEADRIP algorithm applied to the IMU-instrumented sock recordings and the Mocap system, and between (**b**) the Mobility Lab system and the Mocap system during two-minute treadmill walking at different speeds in PsA patients (n = 21, one session). Cadence (CAD), gait cycle duration (GCD), gait speed (GS), stride length (SL), and Motion capture system (Mocap). The solid lines indicate the mean test-retest differences (bias) and the dashed lines indicate the upper and lower 95% limits of agreement (1.96 SD of the bias). Dashed green, red, and blue squares represent the observations for slow, normal, and fast speeds, respectively.

- Treadmill walking:

Bland–Altman plots showing the mean differences and the limits of agreement of the TEADRIP estimations from the IMU-instrumented sock recordings and the estimations from the ML system of CAD, GCD, GS, and SL for all speeds combined for HP (Figure 2) and PsA patients (Figure 3), are presented below. Figure 2 includes the data from sessions one and two and the observations (50 observations in total). Although some outliers are present, the results show a good agreement between the two IMU-based systems and the gold standard as the biases are close to zero in the HP and PsA patients. No systematic biases were observed for either system in estimating the spatio-temporal parameters at slow, normal, and fast speeds in both groups.

In HP, the biases of the temporal parameter estimations from the IMU-instrumented sock were higher than those obtained from the ML system while those of the spatial parameter estimations were lower. In PsA patients, the biases of the temporal and spatial parameters from the IMU-instrumented socks were both lower than those obtained from ML. However, 95% limits of agreement around the difference between the motion capture system and the ML system estimates were narrower than those between the motion capture system and those based on the IMU-instrumented sock.

**Figure 4:**
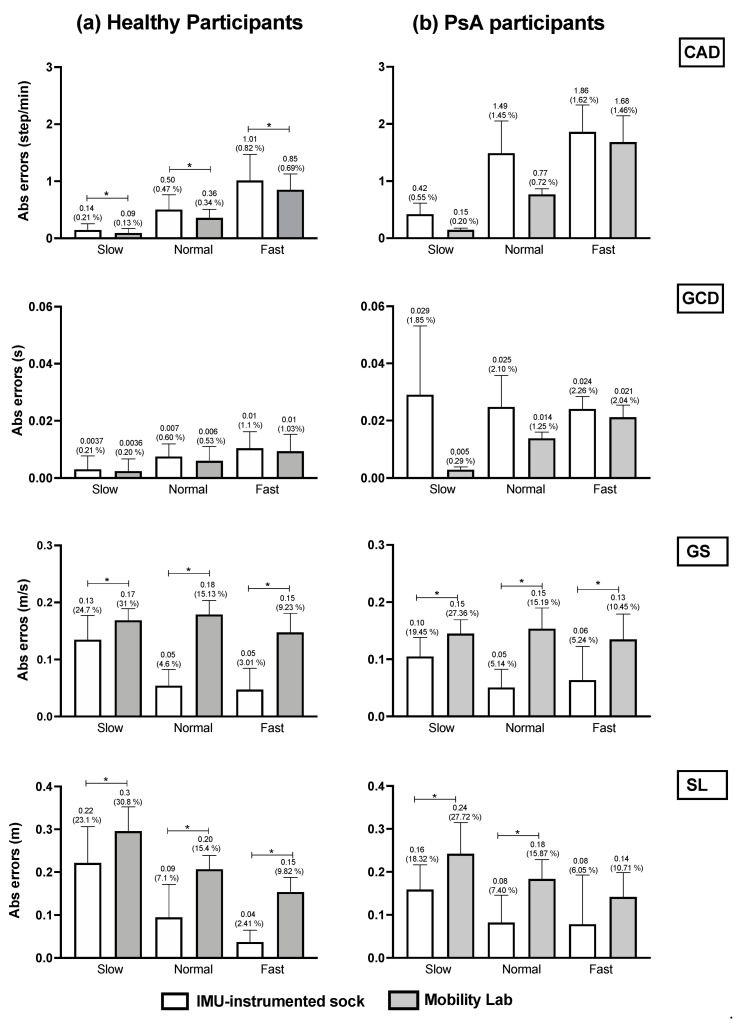
Accuracy comparison between the TEADRIP algorithm applied to the IMU-instrumented sock recordings and the Mobility Lab system estimations of the spatio-temporal parameters in (**a**) healthy and (**b**) PsA participants. Mean absolute errors and relative errors (%) in CAD, GCD, GS, and SL estimations obtained from the IMU-instrumented sock and the Mobility Lab system during 2 min treadmill walking at different speeds. Cadence (CAD), gait cycle duration (GCD), gait speed (GS), stride length (SL), Psoriasic arthritis (PsA). *: *p* < 0.05.

We compared the mean absolute errors (MAE) derived from the IMU-instrumented sock to those from the ML system in HP and PsA patients using a two-way repeated-measures ANOVA (Figure 4). The results presented in Figure 4a,b shows that compared to the motion capture system, the MAE and the relative errors in the estimations of CAD from the TEADRIP applied to the IMU-instrumented sock were <1 step/min (0.82%) in HP, and <1.86 step/min (1.62%) in PsA patients, respectively, across all walking speeds. For GCD, the MAE and relative errors were <0.01 s (1.1%) and <0.033 s (2.26%) in HP and PsA patients, respectively. The findings also show slightly but statistically significant higher errors in the estimations of CAD with the IMU-instrumented sock than with the ML system for all the speeds in HP. The same results were obtained for GCD, but the differences were not statistically significant. GS and SL estimated with the TEADRIP applied to the IMU-instrumented sock recordings were more accurate than those obtained with the ML system in both healthy and PsA participants, as the mean absolute errors were statistically lower except for SL at a fast speed in the PsA group, where no differences between the two systems were found. There were no significant interaction effects between the groups and systems on MAE across all variables and speeds. The precision (intersession reliability) was assessed only in HP. The intraclass correlation coefficients (ICCs) for intersession reliability are presented in Table 2. At slow speeds and for all the parameters, both systems exhibited moderate to good reliability: 0.625 < ICC < 0.798. At normal speeds, ICC was good except for GS and SL obtained from the IMU-instrumented sock recordings. Excellent reliability coefficients were observed at fast speeds for both systems when measuring CAD, GCD, GS, and SL.

**Figure 5:**
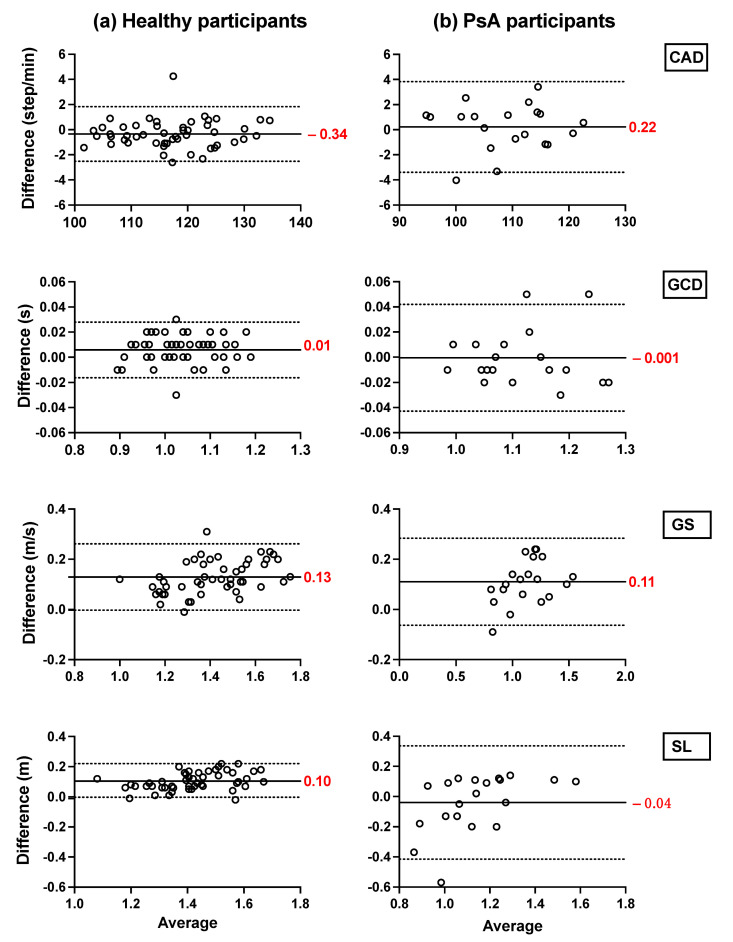
Agreement between the TEADRIP applied to the IMU-instrumented sock recordings and the Mobility Lab system. Bland–Altman plots of the differences between the IMU-instrumented sock and the Mobility Lab system in the spatio-temporal estimations measured for overground walking in (**a**) healthy and (**b**) PsA participants. The solid lines indicate the mean test-retest differences (bias) and the dashed lines indicate the upper and lower 95% limits of agreement (1.96 SD of the bias).

**Figure 6:**
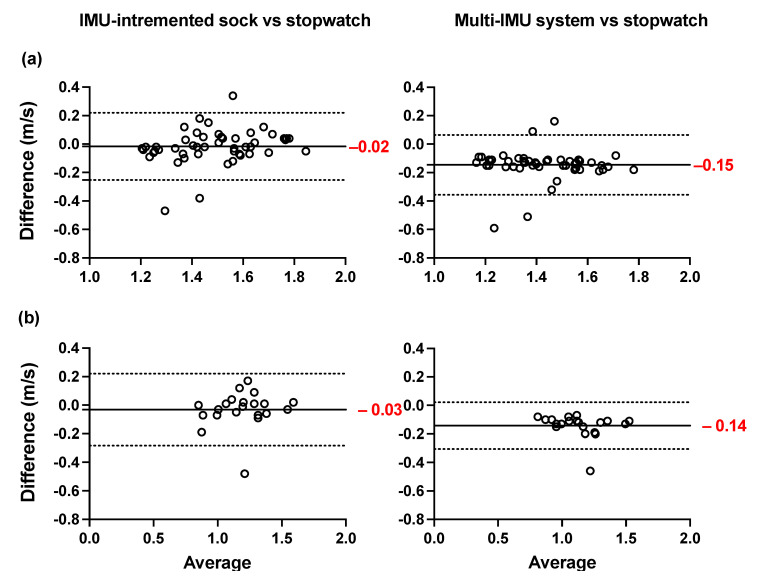
Agreement and Bland–Altman plots of the differences between the TEADRIP algorithm applied to the IMU-instrumented sock recordings and the stopwatch, and between the Mobility Lab system and the stopwatch in GS estimations for overground walking in (**a**) healthy and (**b**) participants.

- Overground walking:

The Bland–Altman plots of the averages and differences between the estimations of the spatio-temporal parameters obtained from the IMU-instrumented sock and those obtained from the ML system are presented in Figure 5a,b. The results show a good agreement between the two systems across all parameters when healthy and PsA participants performed the 10MWT at a self-selected comfortable speed. The biases were lower in PsA patients (Figure 5b) compared to HP (Figure 5a). However, the limits of agreement were narrower in HP. The ICCs presented in Table 3 show good to excellent intersession reliability for all the measurements obtained with the two systems (Table 3). In the same table, the results show a good but lower reliability coefficient (ICC = 0.77) of the stopwatch measurement of gait speed. The Bland–Altman plots of the agreement between the two systems and the 10MWT in healthy and PsA participants (Figure 6a,b) show a good agreement between both systems and the values of GS obtained from the stopwatch. However, the estimation of GS obtained by the IMU-instrumented sock had a lower bias than that from the ML system (−0.02 vs. 0.15 in HP, and −0.03 vs. −0.14 in PsA participants, *p* < 0.0001).

## 4. Discussion

In this study, we assessed the accuracy and precision of a new wearable system in healthy participants and PsA patients with foot impairments. The proposed wearable system applies the TEADRIP algorithm to the recordings of an IMU-instrumented sock (Sensoria^®^) to obtain estimations of the spatio-temporal gait parameters in clinical and eventually, in free-living conditions. The system was validated against a motion capture system that is considered as a gold standard (Optitrack^®^) for treadmill walking and compared to a wearable multi-IMU-based system (Mobility Lab, APDM^®^) for overground walking. We also assessed the reliability of the TEADRIP algorithm applied to the IMU-instrumented sock recordings and the Mobility Lab over two measurement sessions. For treadmill walking, our findings showed a good agreement between the estimations of the spatio-temporal gait parameters obtained from the motion capture system and those obtained from the IMU-instrumented sock in healthy and PsA participants. A good agreement was also found between the spatio-temporal estimations by the Mobility lab system and those estimated by the IMU-instrumented sock for overground walking in both groups. Although the spatio-temporal parameters were significantly different between the group (i.e., a different gait pattern), there were no interaction effects between group and system on MAE which means that the effects of the systems on MAE were the same in healthy and PsA participants. Compared to the motion capture system, the MAE and relative errors in CAD estimated by the IMU-instrumented sock in healthy and PsA participants were ≤1 step/min (0.8%) and <1.86 steps/min (1.62%), respectively, across all walking speeds. For GCD, the MAE and relative errors were <0.01 s (1.1%) and <0.03 s (2.26%) in healthy and PsA participants, respectively, which is an excellent result. In a previous study, Donath et al. demonstrated similar relative errors of 1.5% in CAD estimations and lower errors (0.8%) in GCD estimations obtained from a foot-mounted IMU (Rehagait system) [17]. Salarian et al. estimated GCD with an error of 2% and in the study of Trojaniello et al., the errors were about 1% for the same variable. Rampp et al. showed a similar MAE in GCD estimations (0.03 s) compared to those demonstrated in our PsA group when they compared a foot-mounted inertial sensor system (Shimmer 2R IMU placed laterally on the shoe below each ankle joint) against the GAITRite system in a sample of 101 geriatric patients for normal walking [18].

For gait speed estimation, the TEADRIP algorithm applied to the IMU-instrumented sock performed better than ML in terms of accuracy in both healthy and PsA participants. However, we reported large errors especially at slow walking speeds (relative errors: for the IMU-instrumented sock and ML were 24.7% and 31% in HP, and 19.45% and 27.36% in the PsA group, respectively). This result could be explained by the very low speed we administered (0.45 m/s) that might have been slow enough to drastically alter the kinematics of the lower limbs making the computation of the spatial parameters from the IMU difficult. Moreover, the absolute errors found in the present study were below the minimal clinically important difference (MCID) reported in [30] for gait speed (MCID = 0.20 m/s). We also found larger errors in the estimated values of SL (2.41% < relative errors < 23.1% in HP and 6.05 < relative errors < 18.32 in PsA participants) compared with the study of Trojaniello et al. where the relative errors in stride length estimation were <3% [22]. Rampp et al., also found a smaller MAE in SL estimations (6.26 cm compared to 22 cm, 9 cm, and 4 cm in HP, and 16 cm, 8 cm, and 8 cm in PsA participants at slow, normal, and fast walking speeds as found in our study) [18]. The improved accuracy achieved in the latter studies may be related to the fact that they used two IMUs versus one monolateral IMU in our study. Besides, the low sampling frequency we used in this study may have affected the resolution of GE detection which in turn could have affected the estimation of the spatio-temporal gait parameters. Since our eventual aim is to use the IMU-instrumented sock for free-living and long-term recordings, we chose to set the instrumented sock’s IMUs at a low sampling frequency for usability reasons and to ensure a longer battery life. The differences could also be explained by the different experimental conditions used in our study, as we only assessed the accuracy of the IMU-instrumented sock for treadmill walking at imposed walking speeds while in the other studies, it was assessed for overground walking. Treadmill walking is known to alter foot biomechanics including foot striking patterns which could make it harder for inertial sensors to estimate specific gait parameters accurately [31,32].

With regards to reliability, the ICCs for intersession reliability showed that the ML system exhibited better reliability than the IMU-instrumented sock especially for the computation of spatial parameters when the participants walked on the treadmill at slow and normal walking speeds. The slow walking speed we administered in this study was extremely low (0.45 m/s) and may have caused the participants to undertake different compensatory walking strategies at each testing session. As the ML system uses six IMUs, this could play a major role in the better reliability achieved with the latter system. Moreover, the Mobility Lab system excludes extreme values which could explain the better reliability achieved with this system.

During overground walking (10MWT), there was a good agreement between the IMU-instrumented sock and the Mobility Lab across all the parameters in both groups. There was also a good agreement between both systems and the stopwatch estimations of gait speed. However, errors in the GS estimations from the IMU-instrumented sock were lower than those from the Mobility Lab. Moreover, the TEADRIP applied to the IMU-instrumented sock exhibited a better reliability in estimating the spatial parameters and the ICCs were comparable with those obtained with the ML system. In a previous study, the authors found higher ICCs than those obtained in this study, but using different configurations of the ML system, one based on foot-mounted IMUs and the other based on ankle IMUs [11]. However, the authors did not report the confidence intervals around the ICCs. Moreover, they did not specify if their data were normally distributed. If not, this may have caused an overestimation of the ICC values.

Overall, our findings support the claim that ankle-mounted IMUs are at least as good as foot-mounted IMUs for estimating the temporal gait parameters in healthy and PsA patients with moderate to severe foot pain. In the previous studies, ankle IMUs have always been attached above the ankle with straps or tapes which is not practical for use in free-living conditions, and does not guarantee a consistent placement of the unit over time. The IMU-instrumented sock offers an alternative which is easy and comfortable to wear in daily life.

There are some limitations to this study. First, we assessed the validity of the IMU-instrumented sock versus the motion capture system for treadmill walking and used a different reference system for overground walking. However, using the treadmill enabled us to analyze a large number of steps, and the two reference systems were compared on treadmill trials to minimize this impact. Second, only averaged spatio-temporal parameters were estimated and compared between the systems while it would be relevant to consider the step-by-step accuracy. Finally, we did not include turns in our experimental settings; only straight forward walking was considered. Nevertheless, free-living walking includes some deviations such as turns and stair climbing. Thus, it would be interesting to validate the IMU-instrumented sock system for these conditions.

## 5. Conclusions

We proposed a system to determine the spatio-temporal parameters of gait based on an easy-to-use non-cumbersome sock system that could be worn with all shoe types. These characteristics are very important for a wearable system to be used in a free-living environment and for a long period of time. The proposed system has been proven to be accurate for the estimation of temporal gait parameters and had better accuracy than a system commonly used in clinical applications (Mobility Lab) in estimating spatial parameters. The IMU-instrumented sock systems could potentially be used for the estimation of the spatio-temporal parameters of gait in clinical settings and in free-living conditions, but the data should be interpreted with spatial precautions in patients with severely reduced gait speed. Further studies are needed to validate the system in different conditions and populations.

## Figures and Tables

**Table 1:** Descriptions of the spatio-temporal parameters and the comparison between healthy and PsA patients.

Variables	Systems	Healthy Participants	PsA Patients	*p*
		Mean ± SD	Mean ± SD	
CAD (steps/min)	IMU-instrumented sock	116.45 ± 7.25	109.05 ± 7.79	0.002
	Mobility Lab	117.18 ± 7.23	107.14 ± 10.94	0.001
GCD (s)	IMU-instrumented sock	1.04 ± 0.06	1.11 ± 0.08	0.002
	Mobility Lab	1.03 ± 0.07	1.14 ± 0.14	0.003
GS (m/s)	IMU-instrumented sock	1.46 ± 0.16	1.18 ± 0.22	<0.001
	Mobility Lab	1.34 ± 0.14	1.04 ± 0.23	<0.001
SL (m)	IMU-instrumented sock	1.46 ± 0.13	1.13 ± 0.24	<0.001
	Mobility Lab	1.37 ± 0.12	1.14 ± 0.18	<0.001

Standard deviation (SD), Cadence (CAD), gait cycle duration (GCD), gait speed (GS), and stride length (SL).

**Table 2:** Intersession reliability of the spatio-temporal parameters estimated by the TEADRIP algorithm applied to the IMU-instrumented sock recordings and those estimated by the Mobility Lab system for treadmill walking in HP. Intra Class Correlation Coefficient (ICCs) and their 95% CI for the treadmill 2MWT at slow, normal, and fast walking speeds.

Parameter	System		Speed	
		**Slow (0.45 m/s)**	**Normal (1.12 m/s)**	**Fast (1.6 m/s)**
CAD	IMU-instrumented sock	0.712 (0.34 to 0.88)	0.88 (0.72 to 0.95)	0.934 (0.85 to 0.97)
	Mobility Lab	0.734 (0.40 to 0.88)	0.88 (0.7 to 0.95)	0.938 (0.86 to 0.97)
GCD	IMU-instrumented sock	0.755 (0.44 to 0.9)	0.902 (0.76 to 0.96)	0.936 (0.85 to 0.0.73)
	Mobility Lab	0.778 (0.5 to 0.9)	0.891 (0.725 to 0.95)	0.935 (0.85 to 0.972)
GS	IMU-instrumented sock	0.625 (0.14 to 0.84)	0.657 (0.2 to 0.85)	0.719 (0.4 to 0.88)
	Mobility Lab	0.746 (0.41 to 0.089)	0.835 (0.62 to 0.93)	0.757 (0.44 to 0.89)
SL	IMU-instrumented sock	0.633 (0.2 to 0.84)	0.681 (0.5 to 0.92)	0.914 (0.757 to 0.97)
	Mobility Lab	0.798 (0.54 to 0.91)	0.874 (0.7 to 0.95)	0.916 (0.8 to 0.97)

Cadence (CAD), gait cycle duration (GCD), gait speed (GS), and stride length (SL). Data is presented as Intraclass correlation coefficients (ICCs) and lower and upper 95% confidence limits.

**Table 3:** Intersession reliability of the spatio-temporal parameters estimated by the TEADRIP algorithm applied to the IMU-instrumented sock and those estimated by the Mobility Lab system for overground walking. Intra Class Correlation Coefficient (ICCs) and their 95% CI for the 10MWT.

Parameter	System	Mean (CI)
CAD (STEP/MIN)	IMU-instrumented sock	0.918 (0.817 to 0.964)
	Mobility Lab	0.927 (0.834 to 9.68)
GCD (S)	IMU-instrumented sock	0.892 (0.754 to 0.952)
	Mobility Lab	0.903 (0.779 to 0.957)
GS (M/S)	IMU-instrumented sock	0.802 (0.558 to 0.912)
	Mobility Lab	0.832 (0.617 to 0.926)
	IMU-instrumented sock	0.772 (0.492 to 0.899)
SL (M)	IMU-instrumented sock	0.830 (0.621 to 0.925)
	Mobility Lab	0.827 (0.694 to 0.924)

Cadence (CAD), gait cycle duration (GCD), gait speed (GS), and stride length (SL).

## Data Availability

The data presented in this study are available on request from the corresponding author. The data are not publicly available due to privacy and ethical restrictions.
